# Risk of Parkinson’s disease after human papillomavirus infection: a nationwide cohort study

**DOI:** 10.1186/s12916-025-04444-w

**Published:** 2025-11-07

**Authors:** Tien-Wei Hsu, Chih-Wei Hsu, Yu-Chen Kao, Shih-Jen Tsai, Ya-Mei Bai, Tung-Ping Su, Tzeng-Ji Chen, Mu-Hong Chen, Chih-Sung Liang

**Affiliations:** 1 Department of Psychiatry, E-DA Dachang Hospital, I-Shou University, Kaohsiung, Taiwan; 2 Department of Psychiatry, E-DA Hospital, I-Shou University, Kaohsiung, Taiwan; 3 School of Medicine, College of Medicine, I-Shou University, Kaohsiung, Taiwan; 4 Department of Psychiatry, Kaohsiung Chang Gung Memorial Hospital and, Chang Gung University College of Medicine, Kaohsiung, Taiwan; 5 Department of Psychiatry, Tri-Service General Hospital, National Defense Medical Center, Taipei, Taiwan; 6 Department of Psychiatry, Beitou Branch, Tri-Service General Hospital, Taipei, Taiwan; 7 Department of Psychiatry, Taipei Veterans General Hospital, Taipei, Taiwan; 8 Department of Psychiatry, College of Medicine, National Yang Ming Chiao Tung University, Taipei, Taiwan; 9 Department of Psychiatry, Cheng Hsin General Hospital, Taipei, Taiwan; 10 Department of Family Medicine, Taipei Veterans General Hospital, Taipei, Taiwan; 11 Institute of Hospital and Health Care Administration, National Yang Ming Chiao Tung University, Taipei, Taiwan; 12 Department of Family Medicine, Taipei Veterans General Hospital, Hsinchu Branch, Hsinchu, Taiwan

**Keywords:** Human papillomavirus, Parkinson’s disease, Risk factors, Longitudinal cohort study, Accelerated failure time

## Abstract

**Background:**

Little is known about the association between human papillomavirus (HPV) infection and Parkinson’s disease (PD). This study aimed to explore the risk of incident PD following HPV infection.

**Methods:**

Patients 40 years or older with HPV infection diagnosed between 1996 and 2013 were included in the case group and were 1:4 matched with controls from the Taiwan National Health Insurance Research Database. A second matched control group of patients with acute respiratory infection (ARI) was also included. The outcome was incident PD, as determined using Cox proportional hazards.

**Results:**

The study population included 80,318 patients with HPV infection, 322,952 non-HPV infection matched controls, and 80,318 ARI infection matched controls. There were 456 incident PD cases in the HPV group (0.84 per 1000 person-year), 2499 in the control group (0.43 per 1000 person-year), and 655 in the ARI control group (0.45 per 1000 person-year). After adjustment for confounding factors, patients with HPV infection were associated with a 1.25-fold higher risk of incident PD (hazard ratio, 1.25, 95% confidence interval, 1.13 to 1.39) than controls. This finding remains consistent when comparing to the ARI control group and in sensitivity analyses. Stratified by sex, only male patients with HPV infection had a 1.48-fold higher risk of PD compared with the control group (1.28 to 1.71) and a 1.31-fold higher risk of PD compared with the ARI control group (1.09 to 1.59); however, no significant associations were observed in the female patients with HPV.

**Conclusions:**

This study suggests an increased risk of incident PD in patients with HPV infection, especially for males.

**Supplementary Information:**

The online version contains supplementary material available at 10.1186/s12916-025-04444-w.

## Background

Parkinson’s disease (PD) is a neurodegenerative disorder affecting over 8.5 million people globally [1]. It is characterized by motor symptoms, including tremors, rigidity, and bradykinesia, and non-motor symptoms, including olfactory dysfunction, cognitive impairment, psychiatric symptoms, sleep disorders, and autonomic dysfunction [2]. Importantly, PD is associated with increased economic and caregiver burden, decreased overall quality of life, and a higher mortality [3]. The crucial pathophysiology of PD includes loss of dopaminergic neurons within the substantia nigra and aggregated α-synuclein (Lewy pathology), possibly due to genetic (e.g., *SNCA*,* LRRK2*,* GBA*) and environmental factors (e.g., pesticide exposure, prior head injury, beta-blocker use) [2].

Viral infection is one of the etiologies of PD [4]. Epidemiological studies have reported an increased risk of subsequent PD in patients infected with the hepatitis C virus [5], herpes virus [6], and Dengue virus infection [7]. Parkinsonism can emerge during other viral infections, such as Coxsackievirus and Flaviviruses (e.g., Japanese encephalitis and West Nile virus), showing a preference for basal ganglia involvement. Possible mechanisms include direct infection of the central nervous system, promotion of chronic inflammation, and the production of high levels of cytokines that can breach the blood–brain barrier. This process induces cellular oxidative stress, alters neurotransmitter systems, and leads to the misfolding and aggregation of pathological proteins [4, 8].

The human papillomavirus (HPV) is one of the most prevalent sexually transmitted deoxyribonucleic acid (DNA) viruses worldwide. Approximately 120 subtypes of HPV have been identified in various studies, approximately 40 of which result in infectious cycles in the host [9]. Most research has focused on the impact of HPV infection on mucosal and epithelial tissues, but not on the central nervous system (CNS). Low-risk types, such as HPV types 6 and 11, are associated with anogenital warts or low-grade changes in cervical cells [10, 11]. High-risk groups, especially types 16 and 18, are associated with cervical cancer, squamous cell carcinoma, and head and neck cancers [10, 12]. Importantly, preliminary research suggests that HPV infection is associated with Alzheimer's Disease through interactions with other pathogens and cytokines [13]. However, to date, no studies have examined the association between HPV infection and PD, a major neurodegenerative disease.

The objective of the current study was to examine the risk of incident PD after an HPV infection diagnosis using data from a nationwide cohort study. We hypothesized that patients with HPV infection would have an increased risk of subsequent PD compared with those without HPV infection.

## Methods

### Data source

The Taiwan National Health Insurance (NHI) program, established in 1995, is a universal single-payer system that provides compulsory health insurance to almost all residents of Taiwan; its coverage rate was approximately 99.7% at the end of 2013. The Taiwan National Health Insurance Research Database (NHIRD) is audited and released by the National Health Research Institutes for scientific study purposes. Individual medical records included in the NHIRD are anonymously maintained to protect patient privacy. The International Classification of Diseases, Ninth Revision, Clinical Modification (ICD-9-CM) codes are used for disease diagnosis. The NHIRD has been used extensively in many epidemiologic studies in Taiwan [14–17]. Taipei Veterans General Hospital’s institutional review board approved the study protocol and waived the requirement for informed consent since this investigation used de-identified data and no human subjects’ contact was required.

### Inclusion criteria for individuals with HPV infection and the control group

**Fig. 1:**
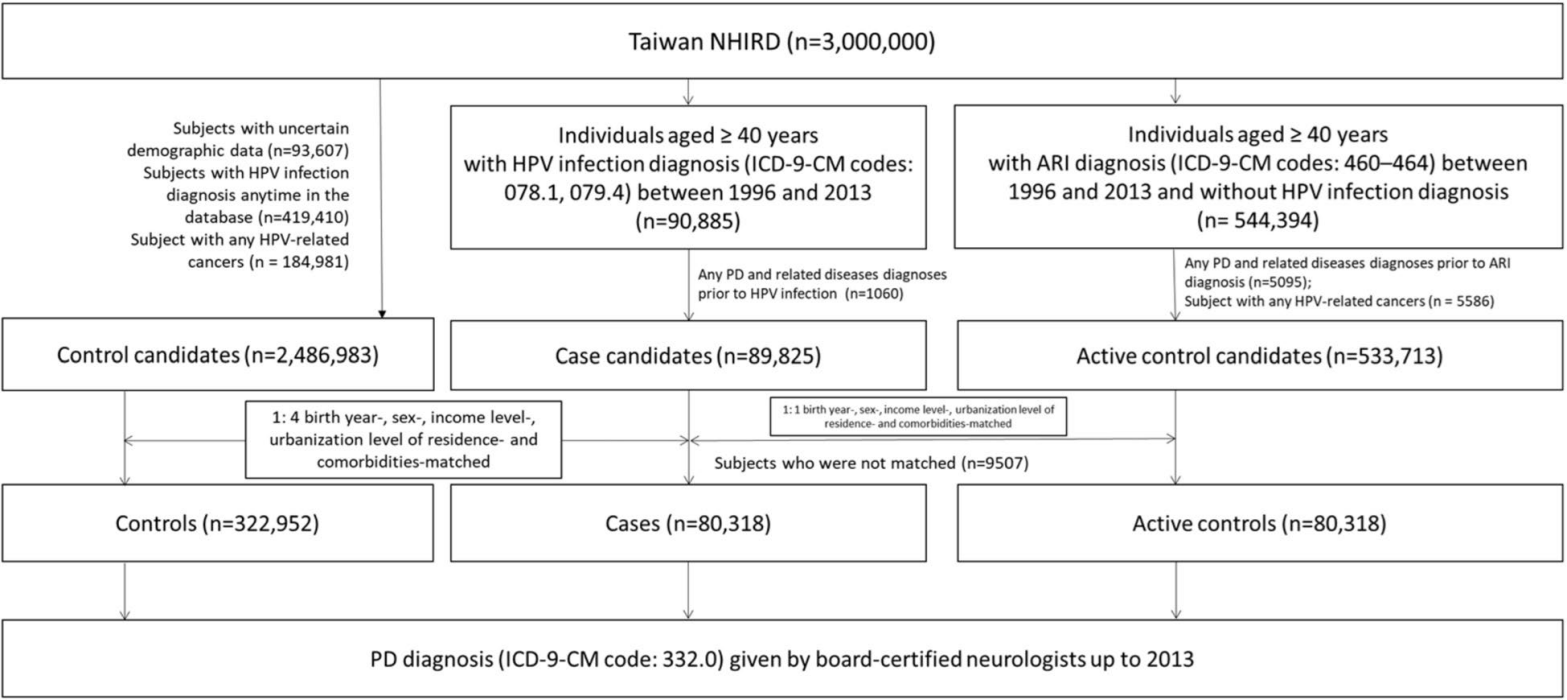
Flowchart of population selection. ARI, acute respiratory infection; HPV, human papillomavirus; ICD-9-CM, International Classification of Diseases, 9th Revision, Clinical Modification; NHIRD, The Taiwan National Health Insurance Research Database; PD, Parkinson’s disease

Individuals aged ≥ 40 years who had a diagnosis of HPV infection (ICD-9-CM codes: 078.1, 079.4) given by board-certified internal medicine physicians, infectious disease physicians, urologists, colorectal physicians, otolaryngologists, obstetricians and gynecologists, and dermatologists between January 1, 1996, and December 31, 2013, at least twice and had no history of PD and related diseases (ICD-9-CM code: 332.x) before enrollment were included as the HPV infection cohort. We randomly selected two control groups from the NHIRD based on the same time point as the index date of HPV infection diagnosis for their corresponding cases using the following procedures. In the first control group, exact matching was performed to match this cohort in a 1:4 fashion to candidate controls who had no diagnosis of HPV infection anytime in the database based on birth year, sex, medical and mental comorbidities, income level, and urbanization level of residence (levels 1–3, most to least urbanized), a proxy for healthcare availability in Taiwan [18]. We also performed exact matching in a second control group to match this cohort in a 1:1 fashion to candidate controls who had no HPV infection diagnosis in the database, were not in the first control group, and had acute respiratory infections (ARI) (ICD-9-CM codes: 460–464) based on birth year, sex, medical and mental comorbidities, income level, and urbanization level of residence. Figure 1 illustrate the steps in participant selection and follow-up. In order to avoid the immortal time bias and the competing effect of death, all subjects survived at the end of the study (December 31, 2013). To minimized the risk of immortal time bias, we carefully aligning the starting time (time zero) for both cases and controls. Specifically, for each individual diagnosed with HPV infection (case), we selected a matched control on the same calendar day of the HPV diagnosis. This ensured that both groups began follow-up at the same time point, eliminating any potential for a period during which individuals in the case group would need to “survive” to qualify for inclusion. A diagnosis of PD (ICD-9-CM code: 332.0) made by board-certified neurologists at least twice was recorded during the follow-up period (from January 01, 1996, to December 31, 2013). Medical comorbidities included cerebrovascular diseases and traumatic brain injury. Mental comorbidities included depressive disorder, alcohol use disorder, and substance use disorder. Charlson comorbidity index (CCI) consisting of 22 physical conditions was also assessed to determine the systemic health conditions of all enrolled subjects [19].

### Statistical analysis

For between-group comparisons, the *F*-test was used for continuous variables and Pearson’s *X*^2^ test for nominal variables. We applied stratified time-dependent Cox-regression analysis with adjustment for CCI scores to investigate the PD risk between groups. The HPV infection state was treated as a time-dependent variable. Sub-analyses stratified by sex were also performed. Given the insidious onset of PD, two types of sensitivity analyses were performed to validate the results by minimizing under-diagnosis of occult PD at the time of HPV infection diagnosis. In the “exclusion of observation period” model, the first year of observation after the diagnosis of HPV infection was excluded, eliminating all cases of PD diagnosed within the year following HPV infection diagnosis. In the “exclusion of enrollment period” model, only patients diagnosed with HPV before the date December 31, 2008, were included in the analysis; patients with HPV diagnosed after these time points were selectively excluded. Additional sensitivity analysis excluding patients with HPV and HPV-related cancers (cervical, oral, anal, vaginal, penile, vulvar cancers) was also performed. Statistical significance was set at two-tailed *P* ≤ 0.05. Data processing and statistical analyses were performed with SAS (version 9.1, SAS Institute, Cary, NC, USA).

### Data availability

The NHIRD was released and audited by the Department of Health and Bureau of the NHI Program for the purpose of scientific research (https://www.apre.mohw.gov.tw/).

## Results

We identified 80,318 patients previously infected with HPV, 322,952 patients without HPV infection as the first control group, and 80,318 patients who had ARI as the second control group, matched for age, sex, physical comorbidities, mental comorbidities, income, and residence (Table 1). The HPV group had a higher CCI score (mean = 2.11, standard deviation, SD = 1.84) compared with the control group (mean = 1.92, SD = 1.87, *z*-score > 3.89) and the ARI control group (mean = 1.93, SD = 1.85, *z*-score > 3.89).

**Table 1:** Demographic characteristics between patients with HPV infection and controls

		Analysis 2^b^			
	Analysis 1^a^				
	(C) Control group (*n* = 322,952)	(P) Patients with HPV infection (*n* = 80,318)	(A) ARI control group (*n* = 80,318)	P vs. C^a^ *p*-value	P vs. A^b^ *p*-value
Birth year (*n*, %)				> 0.999	> 0.999
< 1940	54,704 (17.0)	13,676 (17.0)	13,676 (17.0)		
1940–1949	55,756 (17.4)	13,939 (17.4)	13,939 (17.4)		
1950–1959	112,604 (35.0)	28,151 (35.0)	28,151 (35.0)		
≧1960	98,208 (30.6)	24,552 (30.6)	24,552 (30.6)		
HPV diagnosis age (years, SD)		54.69 (11.33)			
HPV-related cancers (*n*, %)		1068 (0.1)			
Sex (*n*, %)				> 0.999	> 0.999
Male	36,606 (45.6)	36,606 (45.6)	36,606 (45.6)		
Female	43,712 (54.4)	43,712 (54.4)	43,712 (54.4)		
Medical comorbidities (*n*, %)					
Cerebrovascular diseases	33,616 (10.5)	8404 (10.5)	8404 (10.5)	> 0.999	> 0.999
Traumatic brain injury	7320 (2.3)	1830 (2.3)	1830 (2.3)	> 0.999	> 0.999
Depressive disorder	18,012 (5.6)	4503 (5.6)	4503 (5.6)	> 0.999	> 0.999
Alcohol use disorder	7816 (2.4)	1954 (2.4)	1954 (2.4)	> 0.999	> 0.999
Substance use disorder	8288 (2.6)	2072 (2.6)	2072 (2.6)	> 0.999	> 0.999
CCI scores (SD)	1.92 (1.87)	2.11 (1.84)	1.93 (1.85)	< 0.001^c^	< 0.001^c^
Level of urbanization (*n*, %)				> 0.999	> 0.999
1 (most urbanized)	43,904 (13.7)	10,976 (13.7)	10,976 (13.7)		
2	131,396 (40.9)	32,849 (40.9)	32,849 (40.9)		
3 (most rural)	145,972 (45.4)	36,493 (45.4)	36,493 (45.4)		
Income-related insured amount (*n*, %)				> 0.999	> 0.999
≤ 19,100 NTD/month	129,224 (40.6)	32,767 (40.6)	32,767 (40.6)		
19,001 ~ 42,000 NTD/month	145,316 (45.1)	36,424 (45.1)	36,424 (45.1)		
> 42,000 NTD/month	46,732 (14.3)	11,547 (14.3)	11,547 (14.3)		

*HPV *human papillomavirus, *SD *standard deviation, *CCI *Charlson Comorbidity Index, *NTD *new Taiwan dollars, *ARI *acute respiratory infection

^a^A comparison between the control group (C) and patients with HPV (P)

^b^A comparison between the patients with HPV (P) and the patients with ARI (A)

^c^*z*-score > 3.89, two-tailed *p*-value < 0.001

After adjustment for CCI scores (Table 2), patients with HPV infection were associated with a higher subsequent risk of PD (Hazard ratio, HR = 1.25, 95% confidence interval, CI = 1.13 to 1.39) compared with the control group. When comparing with the ARI control group, patients with HPV infection were still associated with a higher risk of PD (HR = 1.20, 95% CI = 1.04 to 1.38). Stratified by sex, male patients with HPV were still associated with an increased risk of PD, no matter when comparing with the control group (HR = 1.48, 95% CI = 1.28 to 1.71) or the ARI control group (HR = 1.31, 95% CI = 1.09 to 1.59). However, the risk of subsequent PD in female patients with HPV infection did not show a significant difference from the control group (HR = 1.04, 95% CI = 0.89 to 1.22) nor the ARI control group (HR = 1.08, 95% CI = 0.88 to 1.33).

**Table 2:** Risk of Parkinson’s disease between groups

		Parkinson’s disease^a^	
	Events (*n*, 1000 person-year)	HR	95% CI
Analysis 1			
All sample			
Control group	2499 (0.43)	1 (ref)	-
Patients with HPV infection	456 (0.84)	**1.25**	**1.13–1.39**
Male sample			
Control group	1227 (0.47)	1 (ref)	-
Patients with HPV infection	258 (1.04)	**1.48**	**1.28–1.71**
Female sample			
Control group	1272 (0.41)	1 (ref)	-
Patients with HPV infection	198 (0.67)	1.04	0.89–1.22
Analysis 2			
All sample			
ARI control group	655 (0.45)	1 (ref)	
Patients with HPV infection	456 (0.84)	**1.20**	**1.04–1.38**
Male sample			
ARI control group	355 (0.54)	1 (ref)	
Patients with HPV infection	258 (1.04)	**1.31**	**1.09–1.59**
Female sample			
ARI control group	300 (0.38)	1 (ref)	
Patients with HPV infection	198 (0.67)	1.08	0.88–1.33

*HPV *human papillomavirus, *CCI *Charlson Comorbidity Index, *HR *hazard ratio, *CI *confidence interval, *ARI *acute respiratory infection

^a^Stratified time-dependent Cox regression analyses with adjustment of CCI scores

In the sensitivity analyses (Table 3), when comparing with the control group, after the exclusion of the first year observation (HR = 1.25, 95% CI = 1.13 to 1.39). After the exclusion of the first 3-year and 5-year observation, we still had consistent findings. After the exclusion of enrollment after 2009 (HR = 1.26, 95% CI = 1.13 to 1.41) and after the exclusion of patients with HPV-related cancers (HR = 1.27, 95% CI = 1.14 to 1.41), the HPV group still showed an increased risk of subsequent PD. When comparing with the ARI control group, the HPV group had higher risks of PD in all sensitivity test.

**Table 3:** Sensitivity analyses for PD risk

HR (95% CI)	All sample	Excluding the first year observation	Excluding the first 3-year observation	Excluding the first 5-year observation	Excluding those enrolled after 2009	Excluding patients with HPV-related cancers
Analysis 1						
Control group	1 (ref)	1 (ref)	1 (ref)	1 (ref)	1 (ref)	1 (ref)
Patients with HPV infection	**1.25 (1.13–1.39)**	**1.25 (1.13–1.39)**	**1.25 (1.13–1.39)**	**1.26 (1.13–1.40)**	**1.26 (1.13–1.41)**	**1.27 (1.14–1.41)**
Analysis 2						
ARI control group	1 (ref)	1 (ref)	1 (ref)	1 (ref)	1 (ref)	1 (ref)
Patients with HPV infection	**1.20 (1.40–1.38)**	**1.20 (1.04–1.38)**	**1.20 (1.04–1.38)**	**1.23 (1.07–1.41)**	**1.21 (1.04–1.40)**	**1.20 (1.05–1.38)**

*HPV *human papillomavirus, *CCI *Charlson Comorbidity Index, *HR *hazard ratio, *CI *confidence interval, *ARI *acute respiratory infection

Note: stratified time-dependent Cox regression models with adjustment of CCI scores

## Discussion

This nationwide cohort study followed participants for 18 years and showed a clear and robust association between HPV infection and incident PD. After adjustment, we found that patients with HPV infection had a 1.25-fold increased risk of incident PD compared to controls without HPV infection. This finding remains consistent when comparing to the second control group who had ARI and when possible cases of insidious onset of PD are excluded. Stratified by sex, only male patients with HPV infection had a higher risk of subsequent risk of PD, but not female patients. HPV papillomavirus infections are associated with the development of various cancers and other health issues [4, 10]. Our findings suggest that HPV infection may be a risk factor for PD. Therefore, healthcare professionals must emphasize the importance of prevention through education of the general public. Educating individuals about the risks associated with HPV infection and promoting preventive measures can significantly contribute to reducing the incidence of serious HPV-related complications.

Viral infections induce PD through various cellular mechanisms. These infections affect host cell responses, diminishing neuronal function by (i) increasing reactive oxygen species (ROS) through interference with the electron transport system and hindering antioxidant production and (ii) promoting neuroinflammation by enhancing the secretion of proinflammatory cytokines [8]. HPV infections follow a similar pathophysiological process. Expression of the E6 protein increases ROS levels in both HPV-positive and HPV-negative cells [20]. E6 expression also decreases the expression of superoxide dismutase isoform 2 and glutathione peroxidase [20]. During the inflammatory process, pro-inflammatory cytokines act on the endothelium of blood–brain barrier cells, triggering the upregulation of adhesion molecules and recruiting circulating T cells and monocytes [21]. HPV is an immunological modulator, which actively induces Th2 and Th17 pathways, with resultant increased production of interleukin-17 (IL-17), IL-13, and subsequently tumor necrosis factor-α (TNF-α) [22, 23]. Furthermore, elevated serum IL-6, IL-8, IL-1β, and macrophage inflammatory protein-1α (MIP-1α) were also observed in patients with HPV infection [24, 25]. Animal studies have shown that neurotoxin 1-methyl-4-phenyl-1,2,3,6-tetrahydropyridine (MPTP)-treated mice have increased numbers of Th17 cells in the substantia nigra [26, 27]. Clinical studies have shown increased Th17 frequency in the peripheral blood of patients with PD compared to healthy controls [28]. Th17 cells actively participate in nigral neurodegeneration by infiltrating the nigral region, which results in excessive activation of microglial cells [29]. Prolonged TNF-α expression leads to continued microglial activation and recruitment of monocytes/macrophages, ultimately resulting in the death of dopaminergic neurons [30]. In the pathogenesis of PD, elevated expression of interferon-gamma (IFN-γ), IL-6, and IL-1β also indicates the crucial role of the inflammatory response in the brain [31]. The severity of PD is correlated with elevated levels of TNF-α and IL-1β [25].

The HPV E6 oncoprotein, commonly generated by HPV 16 and 18, binds to a cellular protein of 100 kDa, termed E6-associated protein (E6-AP) [32, 33]. Subsequently, the E6/E6AP complex recruits and degrades p53, a tumor suppressor protein, which is a major carcinogenic mechanism employed by HPV [32]. E6-AP, homologous to the E6-AP C-terminus domain family E3 ubiquitin ligase, is a component of Lewy bodies in the post-mortem PD brain [34]. In the absence of E6, E6-AP demonstrates ubiquitin ligase activity and promotes the degradation of α-synuclein [33, 34]. Previous studies have demonstrated that the expression of E6 reduces co-expressed E6AP, both in the high-risk type, HPV 16, and the low-risk type, HPV 11 [35, 36]. Long-term infection by HPV with downregulation of E6AP expression, leading to cumulative α-synuclein, may be another mechanism associated with viral PD. However, HPV is an epitheliotropic pathogen. It may be possible for HPV to affect nerve cells or infect the brain. A clinical observational study screened 50 HPV 16-associated samples (15 cervical, 35 head, and neck cancers) and found that the HPV 16 genome exists in a non-replicating form in neurons and constitutively produces high levels of E6 and E7 proteins. [37] In a non-cancer study, HPV antigen expression was elevated in patients with Rasmussen encephalitis, whereas there were no detectable HPV antigens in control patients. [38] Evidence suggests that HPV infects nerve cells in the brain. Nevertheless, further in vivo studies are required to confirm the hypothesis that E6-AP expression is altered by CNS HPV infection.

The prevalence of Parkinson's Disease (PD) is higher in males than in females, with a male-to-female ratio of approximately 1.2:1 to 1.5:1 [39, 40]. This is also reflected in our results, showing that male HPV patients have a higher risk of PD. In past studies on other viral infections and incident PD, it was also found that males had a significantly higher risk while females did not. For instance, after Dengue infection, males had a 3.51-fold higher incidence of PD (HR: 3.51, 95% CI: 1.76 to 7.00), whereas females showed no significant increase (HR: 1.75, 95% CI: 0.73 to 4.19) [7]. Estrogen might play an important role in neuroprotection from development of PD [41]. Several possible mechanisms between estrogen and anti-PD have been studied, including anti-aggregation and fibril destabilization properties in α-synuclein, decreased inflammatory cytokines, decreased microglia activation, decreased leukocyte CNS entry, decreased apoptosis of neurons, increased dopamine synthesis, and increased expression of neurotrophic factors, such as glial cell line-derived neurotrophic factor (GDNF) and brain-derived neurotrophic factor (BDNF) [41–45]. Nevertheless, we still need further studies to investigate the exact mechanisms underlying the sex difference in risk of PD after HPV infection.

### Limitation

Our study has some limitations. First, HPV infections were identified using ICD-9-CM codes in the administrative claims data. Specific HPV types could not be identified from the database. Second, the cumulative incidence of PD was relatively low in our cohort. There were several possible reasons. Although our follow-up period can extend up to 18 years, this duration may be relatively short for the development of PD in some cases. In addition, we used a relatively strict definition of PD, using only one ICD-9 code (332.0), and excluded other related codes. Third, the NHIRD database lacks genetic data; however, it is important to note that inherited genetic mutations linked to PD are widely acknowledged. These mutations are associated with approximately 3–5% of sporadic cases and 30% of familial cases [2]. Fourth, the HPV vaccine would be an important confounding factor. However, in Taiwan, HPV vaccination was self-funded and not covered by the National Health Insurance before 2018. The data period for this study is from 1996 to 2013; therefore, vaccination data would not be recorded in this dataset. Hormonal contraceptives in the NHIRD are also self-funded, meaning they are not covered by the NHIRD, either. Fifth, lifestyle, pollution exposure, or other viral infections might also influence the development of PD, but such data are unavailable in our database or are too complex to calculate. In addition, while we adjusted our results for the CCI score, which includes various clinical conditions, it remains challenging to assess the individual impact of each disease within the CCI index on PD development. Identical CCI scores may reflect different clinical conditions, potentially resulting in unequal effects on the development of PD.

## Conclusions

Our nationwide longitudinal study showed a clear and clinically significant association between HPV infection and the incidence of PD. The increased risk of incidence of PD further consolidates the need for enhanced vigilance and consideration of populations vulnerable to HPV infection. Public education initiatives are needed to inform people that HPV infection may be a risk factor for incident PD. However, further studies are required to validate the mechanisms underlying this association, especially for the sex difference. These studies could even be extended to explore whether there are common or unique mechanisms among different viruses in relation to viral infections and neurodegenerative diseases. In addition to its role in preventing cancer and genital warts, the HPV vaccine may have a preventive effect on PD. This study provides supplementary support for advocating HPV vaccination in clinical practice.

## Supplementary Information

Additional file 1. STROBE Statement—Checklist of items that should be included in reports of cohort studies.

## Data Availability

The NHIRD was released and audited by the Department of Health and Bureau of the NHI Program for the purpose of scientific research (https://www.apre.mohw.gov.tw/).

## Abbreviations

MPTP: 1-Methyl-4-phenyl-1,2,3,6-tetrahydropyridine
ARI: Acute respiratory infection
BDNF: Brain-derived neurotrophic factor
CNS: Central nervous system
CCI: Charlson comorbidity index
CI: Confidence interval
DNA: Deoxyribonucleic acid
E6-AP: E6-associated protein
GDNF: Glial cell line-derived neurotrophic factor
HR: Hazard ratio
HPV: Human papillomavirus
INF: Interferon
IL: Interleukin
ICD-9-CM: International Classification of Diseases, Ninth Revision, Clinical Modification
MIP: Macrophage inflammatory protein
PD: Parkinson’s disease
ROS: Reactive oxygen species
SD: Standard deviation
NHIRD: Taiwan National Health Insurance Research Database
NHI: Taiwan National Health Insurance
TNF: Tumor necrosis factor

## References

[1] Ou Z, Pan J, Tang S, Duan D, Yu D, Nong H, et al. Global trends in the incidence, prevalence, and years lived with disability of Parkinson’s disease in 204 countries/territories from 1990 to 2019. Front Public Health. 2021;9:776847. 34950630 10.3389/fpubh.2021.776847 PMC8688697

[2] Kalia LV, Lang AE. Parkinson’s disease. Lancet. 2015;386(9996):896–912. 25904081 10.1016/S0140-6736(14)61393-3

[3] Kaltenboeck A, Johnson SJ, Davis MR, Birnbaum HG, Carroll CA, Tarrants ML, et al. Direct costs and survival of Medicare beneficiaries with early and advanced Parkinson’s disease. Parkinsonism Relat Disord. 2012;18(4):321–6. 22177623 10.1016/j.parkreldis.2011.11.015

[4] Smeyne RJ, Noyce AJ, Byrne M, Savica R, Marras C. Infection and risk of Parkinson’s DIsease. J Parkinsons Dis. 2021;11(1):31–43. 33361610 10.3233/JPD-202279 PMC7990414

[5] Wu WY, Kang KH, Chen SL, Chiu SY, Yen AM, Fann JC, et al. Hepatitis C virus infection: a risk factor for Parkinson’s disease. J Viral Hepat. 2015;22(10):784–91. 25608223 10.1111/jvh.12392

[6] Cheng CM, Bai YM, Tsai CF, Tsai SJ, Wu YH, Pan TL, et al. Risk of Parkinson’s disease among patients with herpes zoster: a nationwide longitudinal study. CNS Spectr. 2020;25(6):797–802. 31833827 10.1017/S1092852919001664

[7] Hsu TW, Chu CS, Tsai SJ, Cheng CM, Su TP, Chen TJ, et al. Dengue virus infection and risk of Parkinson’s disease: a nationwide longitudinal study. J Parkinsons Dis. 2022;12(2):679–87. 34864691 10.3233/JPD-212938

[8] Wongchitrat P, Chanmee T, Govitrapong P. Molecular mechanisms associated with neurodegeneration of neurotropic viral infection. Mol Neurobiol. 2023. 10.1007/s12035-023-03761-6. 37946006 10.1007/s12035-023-03761-6 PMC11043213

[9] Tommasino M. The biology of beta human papillomaviruses. Virus Res. 2017;231:128–38. 27856220 10.1016/j.virusres.2016.11.013

[10] Brianti P, De Flammineis E, Mercuri SR. Review of HPV-related diseases and cancers. New Microbiol. 2017;40(2):80–5. 28368072

[11] Forcier M, Musacchio N. An overview of human papillomavirus infection for the dermatologist: disease, diagnosis, management, and prevention. Dermatol Ther. 2010;23(5):458–76. 20868401 10.1111/j.1529-8019.2010.01350.x

[12] Malik S, Sah R, Muhammad K, Waheed Y. Tracking HPV infection, associated cancer development, and recent treatment efforts-a comprehensive review. Vaccines. 2023. 10.3390/vaccines11010102. 36679945 10.3390/vaccines11010102 PMC9860736

[13] Block J. Alzheimer’s disease might depend on enabling pathogens which do not necessarily cross the blood-brain barrier. Med Hypotheses. 2019;125:129–36. 30902141 10.1016/j.mehy.2019.02.044

[14] Chen MH, Hsu JW, Huang KL, Su TP, Li CT, Lin WC, et al. Risk and coaggregation of major psychiatric disorders among first-degree relatives of patients with bipolar disorder: a nationwide population-based study. Psychol Med. 2019;49(14):2397–404. 30415649 10.1017/S003329171800332X

[15] Chen MH, Lan WH, Hsu JW, Huang KL, Su TP, Li CT, et al. Risk of developing type 2 diabetes in adolescents and young adults with autism spectrum disorder: a nationwide longitudinal study. Diabetes Care. 2016;39(5):788–93. 27006513 10.2337/dc15-1807

[16] Cheng CM, Chang WH, Chen MH, Tsai CF, Su TP, Li CT, et al. Co-aggregation of major psychiatric disorders in individuals with first-degree relatives with schizophrenia: a nationwide population-based study. Mol Psychiatry. 2018;23(8):1756–63. 29112198 10.1038/mp.2017.217

[17] Zhang B, Wang HE, Bai YM, Tsai SJ, Su TP, Chen TJ, et al. Inflammatory bowel disease is associated with higher dementia risk: a nationwide longitudinal study. Gut. 2021;70(1):85–91. 32576641 10.1136/gutjnl-2020-320789

[18] Liu CY, Hung YT, Chuang YL, Chen YJ, Weng WS, Liu JS. Incorporating development stratification of Taiwan townships into sampling design of large scale health interview survey. J Health Management (Chin). 2006;4:1–22.

[19] Charlson ME, Pompei P, Ales KL, MacKenzie CR. A new method of classifying prognostic comorbidity in longitudinal studies: development and validation. J Chronic Dis. 1987;40(5):373–83. 3558716 10.1016/0021-9681(87)90171-8

[20] Williams VM, Filippova M, Filippov V, Payne KJ, Duerksen-Hughes P. Human papillomavirus type 16 E6* induces oxidative stress and DNA damage. J Virol. 2014;88(12):6751–61. 24696478 10.1128/JVI.03355-13 PMC4054338

[21] Rochfort KD, Cummins PM. The blood-brain barrier endothelium: a target for pro-inflammatory cytokines. Biochem Soc Trans. 2015;43(4):702–6. 26551716 10.1042/BST20140319

[22] Bosteen MH, Tritsaris K, Hansen AJ, Dissing S. IL-17A potentiates TNFalpha-induced secretion from human endothelial cells and alters barrier functions controlling neutrophils rights of passage. Pflugers Arch. 2014;466(5):961–72. 24072078 10.1007/s00424-013-1354-5 PMC4006128

[23] Singh M, Thakral D, Rishi N, Kar HK, Mitra DK. Functional characterization of CD4 and CD8 T cell responses among human papillomavirus infected patients with ano-genital warts. Virusdisease. 2017;28(2):133–40. 28770238 10.1007/s13337-017-0382-8 PMC5510633

[24] Kemp TJ, Hildesheim A, Garcia-Pineres A, Williams MC, Shearer GM, Rodriguez AC, et al. Elevated systemic levels of inflammatory cytokines in older women with persistent cervical human papillomavirus infection. Cancer Epidemiol Biomarkers Prev. 2010;19(8):1954–9. 20647411 10.1158/1055-9965.EPI-10-0184 PMC2919599

[25] Reale M, Iarlori C, Thomas A, Gambi D, Perfetti B, Di Nicola M, et al. Peripheral cytokines profile in Parkinson’s disease. Brain Behav Immun. 2009;23(1):55–63. 18678243 10.1016/j.bbi.2008.07.003

[26] Liu Z, Huang Y, Cao BB, Qiu YH, Peng YP. Th17 cells induce dopaminergic neuronal death via LFA-1/ICAM-1 interaction in a mouse model of Parkinson’s disease. Mol Neurobiol. 2017;54(10):7762–76. 27844285 10.1007/s12035-016-0249-9

[27] Reynolds AD, Stone DK, Hutter JA, Benner EJ, Mosley RL, Gendelman HE. Regulatory T cells attenuate Th17 cell-mediated nigrostriatal dopaminergic neurodegeneration in a model of Parkinson’s disease. J Immunol. 2010;184(5):2261–71. 20118279 10.4049/jimmunol.0901852 PMC2824790

[28] Storelli E, Cassina N, Rasini E, Marino F, Cosentino M. Do Th17 lymphocytes and IL-17 contribute to Parkinson’s disease? A systematic review of available evidence. Front Neurol. 2019;10:13. 30733703 10.3389/fneur.2019.00013 PMC6353825

[29] Brochard V, Combadiere B, Prigent A, Laouar Y, Perrin A, Beray-Berthat V, et al. Infiltration of CD4+ lymphocytes into the brain contributes to neurodegeneration in a mouse model of Parkinson disease. J Clin Invest. 2009;119(1):182–92. 19104149 10.1172/JCI36470 PMC2613467

[30] De Lella Ezcurra AL, Chertoff M, Ferrari C, Graciarena M, Pitossi F. Chronic expression of low levels of tumor necrosis factor-alpha in the substantia nigra elicits progressive neurodegeneration, delayed motor symptoms and microglia/macrophage activation. Neurobiol Dis. 2010;37(3):630–40. 19969084 10.1016/j.nbd.2009.11.018

[31] Fan K, Li D, Zhang Y, Han C, Liang J, Hou C, et al. The induction of neuronal death by up-regulated microglial cathepsin H in LPS-induced neuroinflammation. J Neuroinflammation. 2015;12:54. 25889123 10.1186/s12974-015-0268-x PMC4379721

[32] Martinez-Zapien D, Ruiz FX, Poirson J, Mitschler A, Ramirez J, Forster A, et al. Structure of the E6/E6AP/p53 complex required for HPV-mediated degradation of p53. Nature. 2016;529(7587):541–5. 26789255 10.1038/nature16481 PMC4853763

[33] Scheffner M, Huibregtse JM, Vierstra RD, Howley PM. The HPV-16 E6 and E6-AP complex functions as a ubiquitin-protein ligase in the ubiquitination of p53. Cell. 1993;75(3):495–505. 8221889 10.1016/0092-8674(93)90384-3

[34] Mulherkar SA, Sharma J, Jana NR. The ubiquitin ligase E6-AP promotes degradation of alpha-synuclein. J Neurochem. 2009;110(6):1955–64. 19645749 10.1111/j.1471-4159.2009.06293.x

[35] Brimer N, Lyons C, Vande Pol SB. Association of E6AP (UBE3A) with human papillomavirus type 11 E6 protein. Virology. 2007;358(2):303–10. 17023019 10.1016/j.virol.2006.08.038 PMC1892534

[36] Talis AL, Huibregtse JM, Howley PM. The role of E6AP in the regulation of p53 protein levels in human papillomavirus (HPV)-positive and HPV-negative cells. J Biol Chem. 1998;273(11):6439–45. 9497376 10.1074/jbc.273.11.6439

[37] Fule T, Mathe M, Suba Z, Csapo Z, Szarvas T, Tatrai P, et al. The presence of human papillomavirus 16 in neural structures and vascular endothelial cells. Virology. 2006;348(2):289–96. 16499942 10.1016/j.virol.2005.12.043

[38] Chen S, Chen S, Guan Y, Zhang Y, Qi X, An J, et al. Elevated expression of human papillomavirus antigen in brain tissue of patients with Rasmussen’s encephalitis. Epilepsy Res. 2016;126:119–25. 27490897 10.1016/j.eplepsyres.2016.07.008

[39] Wong SL, Gilmour H, Ramage-Morin PL. Parkinson’s disease: prevalence, diagnosis and impact. Health Rep. 2014;25(11):10–4. 25408491

[40] Khan AU, Akram M, Daniyal M, Zainab R. Awareness and current knowledge of Parkinson’s disease: a neurodegenerative disorder. Int J Neurosci. 2019;129(1):55–93. 29883227 10.1080/00207454.2018.1486837

[41] Smith KM, Dahodwala N. Sex differences in Parkinson’s disease and other movement disorders. Exp Neurol. 2014;259:44–56. 24681088 10.1016/j.expneurol.2014.03.010

[42] Pozzi S, Benedusi V, Maggi A, Vegeto E. Estrogen action in neuroprotection and brain inflammation. Ann N Y Acad Sci. 2006;1089:302–23. 17261778 10.1196/annals.1386.035

[43] Hirohata M, Ono K, Morinaga A, Ikeda T, Yamada M. Anti-aggregation and fibril-destabilizing effects of sex hormones on alpha-synuclein fibrils in vitro. Exp Neurol. 2009;217(2):434–9. 19289119 10.1016/j.expneurol.2009.03.003

[44] Rodriguez-Navarro JA, Solano RM, Casarejos MJ, Gomez A, Perucho J, de Yebenes JG, et al. Gender differences and estrogen effects in parkin null mice. J Neurochem. 2008;106(5):2143–57. 18643794 10.1111/j.1471-4159.2008.05569.x

[45] Goodenough S, Schleusner D, Pietrzik C, Skutella T, Behl C. Glycogen synthase kinase 3beta links neuroprotection by 17beta-estradiol to key Alzheimer processes. Neuroscience. 2005;132(3):581–9. 15837120 10.1016/j.neuroscience.2004.12.029

